# Microbiological Analysis Conducted on Raw Milk Collected During Official Sampling in Liguria (North-West Italy) over a Ten-Year Period (2014–2023)

**DOI:** 10.3390/ani15020286

**Published:** 2025-01-20

**Authors:** Sara Antonia Chiarlone, Andrea Gori, Serena Ravetta, Andrea Armani, Lisa Guardone, Francesca Pedonese, Salvatore Bavetta, Caterina Fiannacca, Nicola Pussini, Cristiana Maurella, Elisabetta Razzuoli

**Affiliations:** 1 Section of Ponente Ligure, Istituto Zooprofilattico Sperimentale del Piemonte, Liguria e Valle d’Aosta, Via Martiri 6, 17056 Savona, SV, Italy; sara.chiarlone@izsplv.it (S.A.C.); nicola.pussini@izsplv.it (N.P.); 2 Department of Veterinary Sciences, University of Pisa, Viale delle Piagge 2, 56124 Pisa, PI, Italy; lisa.guardone@unipi.it (L.G.); francesca.pedonese@unipi.it (F.P.); 3 Section of Genova e Portualità Marittima, Istituto Zooprofilattico Sperimentale del Piemonte, Liguria e Valle d’Aosta, Piazza Borgo Pila 39/24, 16129 Genoa, GE, Italy; serena.ravetta@izsplv.it (S.R.); elisabetta.razzuoli@izsplv.it (E.R.); 4 ASL3—Azienda Sociosanitaria Ligure 3, Sistema Sanitario Regionale Liguria, Via San Giovanni Battista n. 48, 16154 Genoa, GE, Italy; salvatore.bavetta@asl3.liguria.it (S.B.); caterina.fiannacca@asl3.liguria.it (C.F.); 5 Epidemiologia-Sicurezza Alimentare Istituto Zooprofilattico Sperimentale del Piemonte, Liguria e Valle d’Aosta, Via Bologna 220, 10154 Torino, TO, Italy; cristiana.maurella@izsplv.it

**Keywords:** food safety, *Campylobacter* spp., microbiology, official control, *Salmonella* spp.

## Abstract

Milk has been consumed by humans for thousands of years for its nutritional properties. In recent years, raw milk demand has increased, valued for its authenticity and connection to local traditions. In Italy, the sale of raw milk is allowed exclusively through direct sale from the producing farm to the final consumer, either at the producing farm itself or through vending machines. However, the consumption of raw milk is not without risks. Among these, microbiological ones are relevant. These can lead to severe symptoms, particularly in vulnerable populations. For this reason, although consumers are advised to boil raw milk before consumption, producing farms in Italy are required to meet the microbiological criteria outlined in the Provision of 25 January 2007. In this retrospective study, the results of the analyses performed on 355 raw milk samples collected in Liguria between 2014 and 2023 for the detection of *Campylobacter* spp., *Salmonella* spp., *Listeria monocytogenes*, *Staphylococcus aureus*, and *Escherichia coli* O157 were analysed to better characterise the associated risk for consumers. The samples were collected during official controls by the local veterinary health services at vending machines of seven producing farms. Overall, six samples tested positive for *C. jejuni*, while only one sample tested positive for *Salmonella enterica* subsp. *enterica*, Serovar Veneziana. *Listeria monocytogenes*, *S. aureus*, and *E. coli* O157 were never responsible for non-compliances. Interestingly, three of the six samples positive for *C. jejuni* derived from the same producer. In farms where positive samples were detected, certain structural and/or operational non-compliances were identified. It can be concluded that, although the scenario in question does not present any cause for concern, it is nevertheless essential to implement a series of preventive measures in order to guarantee the safety of raw milk. These measures include the implementation of biosecurity practices, the maintenance of strict hygiene protocols during milking, and the adherence to the cold chain distribution protocol until the final stage of distribution.

## Simple Summary

Milk has been consumed by humans for thousands of years for its nutritional properties. In recent years, raw milk demand has increased, valued for its authenticity and connection to local traditions. However, the consumption of raw milk is not without risks. Among these, microbiological ones are relevant. Although consumers are advised to boil raw milk before consumption, producing farms in Italy are required to meet the microbiological criteria outlined in the Provision of 25 January 2007. In this context, official controls play a crucial role in verifying that farms and raw milk comply with these criteria, safeguarding public health. This study analyzed 355 raw milk samples collected in Liguria between 2014 and 2023. The samples were collected by local veterinary health services from vending machines at seven producing farms. Overall, six samples tested positive for *Campylobacter jejuni*, and one sample was positive for *Salmonella enterica* subsp. *enterica*, Serovar Veneziana. *Listeria monocytogenes*, *Staphylococcus aureus*, or *Escherichia coli* O157 were never responsible for non-compliances. Although these findings suggest a low risk in the analyzed region, preventive measures must be implemented by farms to impede milk contamination by these microorganisms.

## 1. Introduction

Milk has been consumed by humans for thousands of years for its rich nutritional properties [1]. Currently, about 160 million tons of milk are produced in Europe, and of these, 22.3 million tons are for direct consumption [2]. Thermal treatments on milk are generally applied to ensure its safety and stability [3]. Current heat treatments include high temperatures for short time (HTST) pasteurization, ultra-pasteurization, and ultra-high temperature (UHT) processing [4,5,6]. Non-thermal processes such as microfiltration and bactofugation can also be applied and are usually combined with a final pasteurization [4].

In recent years, changes in consumer habits revealed a growing preference for minimally processed foods or, even better, for those not subjected to heat treatments, such as raw milk [7]. This is probably driven by consumers’ perceptions that raw milk sold directly by producers is a more authentic and genuine product, with a strong connection to local traditions and territory [8,9]. Indeed, although the supposed higher nutritional qualities of raw milk compared to pasteurized milk have been scientifically questioned [10,11], the demand for raw milk is increasing all over the world [12,13].

According to the European Union (EU) legislation, raw milk is defined as “milk produced by the secretion of the mammary gland of farmed animals that has not been heated to more than 40 °C or subjected to equivalent treatments” [14]. In the EU, the decision to allow the sale of raw milk is delegated to individual Member States (MS) [14]. Based on the latest data provided by the European Food Safety Authority (EFSA), the sale of raw milk is permitted in Belgium, Denmark, France, Germany, Ireland, Italy, the Netherlands, and parts of the United Kingdom [15].

With regard to the Italian context, the sale of raw milk is permitted exclusively through direct sale from the producing farm to the final consumer. This can occur in two ways: firstly, directly at the producing farm; and secondly, through vending machines, which can be located on the farm premises or even offsite, provided they are within the territory of the province where the farm is located or in neighboring provinces, and that the producer is clearly indicated. More than one producer may utilize the same vending machine; however, it is necessary to indicate daily which producer’s milk is being sold [16]. In 2013, Italy had the largest number of raw milk vending machines in EU, with 1066 units [15]. Despite a decrease in both farms and vending machines authorized to sell raw milk between 2016 and 2019, they remain numerous, with a total of 967 units in 2019 [17,18,19,20].

The consumption of raw milk is not without risks [1]. Among the principal hazards, those of a biological nature, particularly those involving microorganisms, are of particular relevance [21]. Raw milk can therefore be a vehicle, if consumed as it is, for various pathogenic microorganisms that are potentially dangerous to human health, such as *Campylobacter* spp., *Salmonella* spp., *Listeria monocytogenes*, *Staphylococcus aureus*, and Shigatoxin-producing *Escherichia coli* (STEC) [22,23,24,25]. These pathogens are responsible for gastrointestinal infections and other severe illnesses, with outcomes that can be particularly critical, or even fatal, for vulnerable subjects [15]. Between 2007 and 2012, 27 outbreaks in Europe were attributed to the consumption of raw milk [15]. Most of these were caused by *Campylobacter* spp. (21), one by *Salmonella*, and two by STEC [15]. In 2017, *L. monocytogenes* was detected in 2.8% of the 2055 units of ready-to-eat (RTE) milk (including raw milk) tested in Europe. Some of these positives were reported from Italy [26]. In the same year, one MS reported the presence of STEC in 2.5% of the 394 raw milk samples analyzed [26]. Furthermore, between 2018 and 2021, raw milk had the highest percentage of *Campylobacter* spp. contamination (0.90% on 1229 samples) compared to other RTE foods [27].

Consequently, the official controls conducted by the Competent Authority (CA) in accordance with Regulation (EU) 2017/625 [28] are essential for the purpose of verifying that farms and products comply with the current legal criteria and for the protection of public health [29]. In Italy, the Local Health Authority (LHA) is responsible for ensuring that farms apply the general food hygiene and safety standards established by Regulations (EC) 178/2002 [30], 852/2004 [31], and 853/2004 [14], and the national mandatory requirements [29]. Specifically, the Provision of 25 January 2007 requires that raw milk meets specific microbiological criteria at the time of sale. These include a limit of 2000 CFU/mL for *Staphylococcus aureus* and the absence of *Campylobacter* spp., *Salmonella* spp., *Listeria monocytogenes*, and *Escherichia coli* O157 in 25 mL. If these criteria are not met, the sale of raw milk must be suspended until the non-compliance is resolved. In 2019, 5065 official analyses were performed in Italy, from which 29 non-conformities were found, with the highest percentage (1.4%) related to the presence of STEC [20]. In 2020, of the 3842 analyses performed, STEC were detected in 16 samples, *Campylobacter* spp. in 11 samples, coagulase-positive *Staphylococcus* in 4 samples, and *L. monocytogenes* in 1 sample [32]. According to the same Provision of 2007, the LHA also verifies that raw milk vending machines display clear and comprehensive information and that, if equipped with a bottling system, some of this information is also visible on the label (Table S1).

In addition, the LHA verifies that the label includes the statement “product to be consumed after boiling”, written in red and in clearly visible characters. This latter information was made mandatory in 2012 [33,34] after several cases of hemolytic uremic syndrome due to consumption of raw milk contaminated by STEC [35]. Boiling is indeed the sole home method to ensure the safety of raw milk [36].

Considering the number of past outbreaks associated with raw milk consumption, it is crucial to monitor the microbiological safety of this product, particularly in areas where its sale is permitted. Thus, the aim of this retrospective study is to analyse the results of microbiological analyses performed on raw milk collected from vending machines during official sampling in Liguria (North-west Italy) over a ten-year period (2014–2023). Our study could allow not only to better characterize the risk for consumers but also to investigate the possible drivers responsible for non-compliance observed.

## 2. Materials and Methods

### 2.1. Data Analysis

This retrospective study only includes data obtained from the Istituto Zooprofilattico Sperimentale of Piemonte, Liguria, and Valle D’Aosta, Italy (IZSPLV) records on results of official control activities carried out by the LHA in farms. In particular, the results of microbiological analysis of raw milk collected during official sampling from a ten-year period (2014–2023) were analyzed. The samples were collected by the LHA in sterile containers at nine raw milk vending machines in the Genova and Savona districts (Liguria region, North-west Italy) (Table S2). Samples were taken refrigerated to the food control laboratory in Genoa and Savona, district sections of the IZSPLV. Consistent with national and regional legislation [16,37] for food safety criteria, raw milk samples were tested for the presence of *Campylobacter* spp., *Salmonella* spp., *L. monocytogenes*, coagulase-positive *Staphylococcus*, and *Escherichia coli* O157. In the following sections, the methods used for microbiological analysis are described. To be noted that, when referring to an ISO method, the last available version was cited, although samples were processed according to the version in use at the time of the analysis. Raw milk samples that did not meet the microbiological criteria provided by current legislation (Table 1) were considered not compliant. The non-compliances identified at the farm level by LHA and the required corrective actions were also analyzed.

#### 2.1.1. *Campylobacter* spp.

The search for *Campylobacter* spp. was conducted following a first screening step using an enzyme-linked fluorescent assay (ELFA) on a MiniVIDAS^®^ analyzer (bioMèrieux, Marcy l’Etoile, France), and the ELFA MiniVIDAS^®^ *Campylobacter* kit (ELFA CAM) (bioMèrieux, Marcy l’Etoile, France, cat. 30111), a method previously validated for use in dairy products [38]. In case of positivity at the screening phase, the enrichment broth was seeded on plates of modified charcoal cefoperazonedeoxycholate agar (mCCDA) (all cultivation media are internally produced by IZSPLV, unless specified) and Campy Food agar (CFA) (bioMèrieux, Marcy l’Etoile, France) incubated in microaerobic conditions at 41.5 ± 1 °C for 44 ± 4 h. Suspected colonies were then selected from each plate, seeded in Columbia agar (microbiol, Cagliari, Italy), and incubated in microaerophilic conditions at 41.5 ± 1 °C for 24–48 h. These pure cultures were then used to confirm the presence of *Campylobacter* spp. by the following assays: (a) observation of the typical bacteria morphology and motility test at optical microscopy; (b) incubation at 25 ± 1 °C in micro-aerobiosis for 44 ± 4 h; (c) incubation at 41.5 ± 1 °C in aerobiosis for 44 ± 4 h; (d) oxidase test. The presence of *Campylobacter* spp. was confirmed based on positive oxidase reaction, typical motility and morphology, and an absence of growth at 25 °C in micro-aerobiosis and at 41.5 °C in aerobiosis.

#### 2.1.2. *Salmonella* spp.

The search for *Salmonella* spp. was conducted by real-time PCR using the iQ-Check *Salmonella* II PCR Detection Kit (BIO-RAD, iQ-Check Salmonella II PCR Detection Kit) using a CFX96 Touch Real-Time PCR Detection System (Bio-Rad, Hercules, CA, USA). Firstly, an enrichment step was conducted by adding 25 ± 0.2 mL of the sample to 225 ± 5 mL of buffered peptone water (BPW) added with 5% *Salmonella* supplement. The mixture was incubated at 37 ± 1 °C for 16–20 h. After this time, DNA was extracted from 1 mL of the enrichment broth. Subsequent steps were conducted according to the kit instructions. In case of positivity, the samples were submitted to confirmation following ISO 6579-1:2017 [39]. Briefly, 1 mL of the enrichment broth was seeded in Müller-Kauffmann tetrathionate-novobiocin (MKTTn) broth (microbiol, Cagliari, Italy) and incubated at 37 °C for 24 ± 3 h, and 100 µL of the pre-enrichment broth were seeded in Rappaport Vassiliadis Soy (RVS) broth and incubated at 41.5 ± 1 °C for 24 ± 3 h. Then, each enrichment medium was seeded on Xylose Lysine Desoxycholate (XLD) agar and Brilliant Green agar (BGA) and incubated at 37 ± 1 °C for 24 ± 3 h. Typical *Salmonella* spp. colonies appear black in XLD and bright pink in BGA. Suspected colonies (minimum 1, maximum 5) are transferred in nutrient AGAR and incubated at 37 ± 1 °C for 24 ± 3 h. Then confirmatory tests were conducted by using Triple-Sugar Iron agar (TSI) and API-20E (bioMèrieux, Marcy l’Etoile, France). Serotype identification of the confirmed positive colonies was carried out using the standard agglutination method, according to ISO/TR 6579-3: 2014 [40].

#### 2.1.3. *Listeria monocytogenes*

The presence of *L. monocytogenes* was assessed by a first screening step using the ELFA VIDAS^®^ *L. monocytogenes* Xpress Assay (bioMèrieux, Marcy l’Etoile, France), according to the manufacturer’s instructions. The method was previously validated [41]. In case of positivity, the sample and the necessary dilutions for the product under test were prepared and seeded on ALOA (Agar Listeria according to Ottaviani and Agosti) plates (Biolife, Milan, Italy), which were then incubated at 37 ± 1° C for 24–48 h. After incubation, the growth of suspected colonies was evaluated. The colonies of *L. monocytogenes* are green–blue surrounded by an opaque halo. In the presence of colonies with such characteristics, confirmatory tests were conducted, selecting 5 suspicious colonies. The colonies were transferred on TSYEA and incubated at 37 ± 1 °C for 18–24 h. The presence of *L. monocytogenes* was confirmed based on Gram staining, catalase activity, hemolysis test, CAMP test, and carbohydrate utilization by API^®^ *Listeria* (bioMèrieux, Marcy l’Etoile, France).

#### 2.1.4. Coagulase Positive *Staphylococcus*

The search for *Staphylococcus* spp. was conducted according to ISO 6888-2:2021 [42]. The samples were diluted and plated by inclusion on Baird Parker agar base (BPA) added with potassium tellurite solution, bovine fibrinogen, rabbit plasma, and trypsin inhibitor to obtain the complete BPA-RPF medium. Each dilution was seeded on two plates. After solidification, the plates were incubated at 37 ± 1 °C for 18–48 h. After the incubation period, coagulase-positive staphylococci form small black, grey, or even white colonies surrounded by an opaque or cloudy halo of precipitation indicating coagulase activity. The characteristic colonies on each plate containing no more than 100 typical colonies were counted, following the ISO 7218:2007/Amd 1:2013 [43].

#### 2.1.5. *Escherichia coli* O157

The search for *E. coli* O157 was conducted using an ELFA method by using the VIDAS^®^ ECPT kit (bioMèrieux, Marcy l’Etoile, France) on a MiniVIDAS^®^ analyzer (bioMèrieux, Marcy l’Etoile, France). Firstly, 25 ± 0.5 mL of sample were collected sterilely, and 225 ± 5 mL of BPW preheated to 41.5 ± 1 °C were added. Then, 1 mL ± 50 µL of vancomycin + cefixime + cefsulodin solution was added. The sample was then homogenized and incubated at 41.5 ± 1 °C for 15/24 h before the MiniVIDAS^®^ analysis. In case of positivity, an immune-concentration phase with VIDAS^®^ I. C. *E. coli* O157 (bioMèrieux, Marcy l’Etoile, France), followed by seeding on CT-SMAC and SMAC and incubated for 18–24 h at 37 ± 1 °C. The suspected colonies (at least 5), which are transparent or present a pale yellow-brownish color, are seeded on blood AGAR, incubated for 18–24 h at 37 ± 1 °C, and subsequently confirmed by PCR (according to ISO 13136:2012) [44].

### 2.2. Statistical Analyses

Data were collected in an ad hoc database and analyzed with the STATA 18.1 software. A descriptive analysis was conducted to study the occurrence of pathogens. The Fisher’s exact test was applied to test the association between the occurrence of pathogens and the following variables: sampling location, year, month, and season.

## 3. Results and Discussion

### 3.1. Overall Non-Compliant Samples

**Figure 1:**
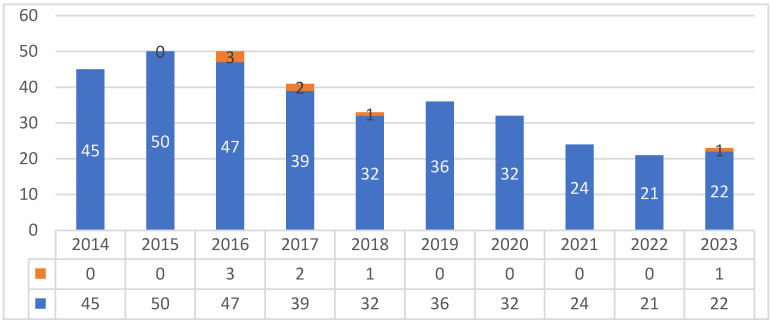
Distribution of negative (in blue) and non-compliant samples (in orange) per year of collection.

In this study, the results of the microbiological analysis of 355 raw milk samples, collected by the LHA during official controls over a ten-year period (from 2014 to 2023), were analyzed. These microbiological criteria (Table 1) must be evaluated through self-monitoring by the food business operators (FBOs) and verified by the LHA by official sampling with a frequency based on risk analysis and using appropriate methodologies and techniques in accordance with Regulation (EU) 2017/625 [28]. Although these are defined as process hygiene criteria [16], exceeding them requires farmers to withdraw the non-compliant milk from the market and suspend the sale until the non-compliance is resolved. Figure 1 illustrates the distribution of samples by year of collection. Overall, seven official samples were non-compliant: six samples tested positive for *C. jejuni* (1.7%), while only one sample tested positive for *Salmonella* spp., identified as *S. enterica* subsp. *enterica*, Serovar Veneziana (0.3%). *L. monocytogenes*, *S. aureus*, and *E. coli* O157 were never detected. No significant correlation was identified between non-compliance and sampling location, nor with the different time dimensions (year, month, season) (*p* > 0.05). Interestingly, three of the six samples positive for *C. jejuni* derived from the same producer (Table 2). The number of samples over the years and the distribution of the non-compliant samples per year are reported in Figure 1.

### 3.2. Campylobacter *spp.*

Although *Campylobacter* spp. infections reported over the past decade have primarily been linked to the consumption of raw or undercooked meat, particularly chicken, raw milk is recognized as a potential vehicle of infection [15,45,46,47,48].

The presence of *Campylobacter* spp. in raw milk is mainly due to fecal contamination, typically occurring during the milking process [49,50]. Indeed, previous studies indicated that *Campylobacter* has been isolated from feces of a variable number of cows in different farms located in Italy [51,52]. Specifically, in the study of Bianchini et al. (2014), *C. jejuni* was isolated from 30.5% of bovine fecal samples (25/82) collected from three dairy farms [51]. While, in the study of Merialdi et al. (2015), 9.2% of fecal samples (26/280) collected from 50 animals tested positive for thermophilic *Campylobacter* [52]. However, the raw milk contamination can happen through the adoption of inappropriate practices, such as poor udder cleaning or inadequate hygiene and maintenance of milking systems [51,53]. Some studies also reported occasional contamination caused by mammary gland infections [54]. Moreover, wild birds can act as reservoirs for *Campylobacter* spp., spreading the bacteria in the environment where dairy cattle live, which may increase the risk of milk indirect contamination [53,55]. In addition to these factors, the microbiological quality of raw milk can also be influenced by the water used to clean and rinse the equipment, particularly after disinfection. Indeed, hard water can promote the formation of deposits in the milking system, on which *Campylobacter* can nest, forming resistant biofilms [53,56]. During official controls on the farms where *C. jejuni* was detected, several structural and operational non-compliances were identified, which may have contributed to milk contamination (Table 2). In farm F, the proximity of the poultry house to the barn and milking area was found to be a factor that could have facilitated contamination of the raw milk. Indeed, poultry is known to be a vehicle of *Campylobacter* [57]. The corrective actions imposed by LHA to the farmer involved relocating the poultry house to a proper distance from the barn and milking area, along with extraordinary cleaning and disinfection of the milking and milk storage areas. In farm A, the same criticism emerged. In response, the farmer started the construction of a new shed for rearing laying hens, away from the barn, and with these new arrangements, no more non-compliances were found. On the contrary, on farm E, in the years 2016–2018, positivity was always found at the raw milk vending machine, due to poor management of the machine itself. The non-compliance was resolved by an extraordinary cleaning and through sanitization of the vending machines and the containers used for transport.

In our study, out of 355 raw milk samples analyzed, 6 (1.7%) tested positive for *C. jejuni*. Other Italian studies available in the literature reported highly variable prevalence rates for *Campylobacter* spp., ranging from 0% up to 12% [50,51,53,58,59,60,61,62,63]. Therefore, our results (1.7%) are in line with previous ones. However, results comparisons among studies are made difficult by several differences among research, such as the sampling point (bulk milk, vending machines, in-line milk filters), the diverse environmental conditions, and the different detection techniques (only conventional culture methods or in association with real-time PCR). Finally, some studies do not specify the *Campylobacter* species analyzed, and others report multiple species without detailing the prevalence of *C. jejuni*, even if present.

Even if the comparison among results obtained during official controls is more feasible, considering that sampling and detection techniques are standardized, the farm characteristics can still influence prevalences [17,18,19,20]. The prevalence of *Campylobacter* spp. found in our study (1.7%) is higher than that observed in official controls (ranging from 0.8% to 1.3%). However, it is crucial to interpret these results in the context of environmental variability and specific farm practices. In this regard, our samples were collected exclusively in the Liguria region, while the data from official controls refer to the entire Italian peninsula. Therefore, it is essential to consider the specific geographical and environmental context of each region, as these factors may influence the transmission and contamination by different pathogens, despite Giacometti et al. (2013) reporting no significant differences among regions [60]. In addition, farm management and milking practices, which can vary considerably from one context to another, may indeed play a crucial role in determining these dynamics [64]. In our study, the farms in which non-compliances were found were all of small size with animals allowed to access an outdoor area next to the barn, often in proximity to the poultry house. These conditions may have increased the risk of indirect contamination of raw milk, contributing to the higher prevalence observed compared to national data. This hypothesis is supported by the fact that, after the relocation of the poultry houses, no further positive results were detected. The role of poultry houses as a potential source of contamination is consistent with the recognized involvement of poultry in the spread of *Campylobacter* spp., and the improvement observed following corrective actions further strengthens this interpretation [57]. Furthermore, it is important to highlight that the proximity between poultry houses, barns, and milking areas observed in the studied farms might represent a structural configuration more common in certain geographical areas, such as Liguria, but not necessarily in other Italian regions. This aspect could partly explain the differences in prevalence observed between our study and national data, suggesting that local management plays a relevant role in determining the risk of contamination.

### 3.3. Salmonella *spp.*

Raw milk is mainly contaminated with *Salmonella* spp. through direct contact with feces of infected animals or from other contaminated environmental sources [65,66]. In some cases, however, infected animals can directly contaminate the milk by excreting the bacteria into it [67]. This poses a relevant risk to public health, as *Salmonella* can cause serious foodborne illnesses in humans, with symptoms such as fever, diarrhea, and abdominal cramps. In severe cases, it can lead to systemic infections or even death [68].

One specific serovar of *Salmonella enterica* subsp. *enterica* that has recently attracted attention is *S.* Veneziana [69]. *S.* Veneziana was in fact not previously considered a relevant cause of human infections [69]. However, its potential pathogenic effects on humans, including the possibility of causing ileitis similar to that seen in Crohn’s disease patients, have long been recognized [70]. Recent studies reported an increase in the prevalence of *S.* Veneziana, particularly in wildlife and surface waters, which could serve as sources of contamination for livestock farms [71,72,73]. Livestock, such as dairy cattle, may be exposed to contaminated water sources or may come into direct contact with wildlife, raising the risk of *Salmonella* transmission [74,75,76].

In our study, one out of 355 raw milk samples (0.3%) tested positive for *S. enterica* subsp. *enterica*, Serovar Veneziana. As for *Campylobacter* spp., it is worth noting that the prevalence observed in our samples falls within the range reported in the Italian literature and official controls, where *Salmonella* prevalences vary from 0 to 1.01% [17,18,19,20,50,53,58,59,60,62]. Overall, the available data suggest that even if *Salmonella* is a relevant pathogen to be monitored, it is not the main biological hazard associated with raw milk consumption. Although *S.* Veneziana was not detected in the only study conducted in Italy in which the serovars were identified [50], it is important to note that this serovar has been documented in several studies conducted in Liguria for different purposes. In particular, Giorda et al. (2014) highlighted the presence of *S.* Veneziana in wild boars and foxes, two species that could act as natural reservoirs of this pathogen [77], while Razzuoli et al. (2017) found its presence in both a human and in surface waters, demonstrating that this pathogen had already been present in analyzed territory [69]. So, although there is no conclusive evidence that the contamination of the raw milk sample analyzed in this study is attributable to direct contact between wildlife or surface waters and the dairy farms from which the raw milk originated, these sources of contamination could represent a potential transmission route. The official controls in farm G, where the positivity for *Salmonella* was detected in 2017, revealed several issues within the farm itself. These included an improper cleaning of the post used during milking and an incorrect straw bedding placement. Another non-compliance identified concerned the access to the barn and the milk storage area, which were not protected by barriers, thus allowing undesirable animals and unauthorized personnel to access it. In fact, wildlife can indirectly contaminate water sources and environments used by cattle through the excretion of infected feces. Furthermore, similar to farms A and F, the proximity of the poultry area to the barn posed a potential risk. In response to these non-compliances, the farmer took several corrective actions. Firstly, extraordinary rat control measures were implemented, and precautions were taken to prevent any direct contact between the cows and the poultry area. To address the access to the barn and the milk storage area, the farmer installed security gates to restrict entry. In addition, the farmer carried out cleaning of the shelter and milk storage area and milking equipment. After these corrective actions, *Salmonella* has not been detected anymore on the farm.

Therefore, even if the prevalence of *Salmonella* in our samples was low, its presence in raw milk sold to consumers highlights the importance of ongoing monitoring and control of *Salmonella* in dairy farms [78]. In fact, even minimal contamination can pose risks to consumers, especially if the milk is not boiled before consumption [36].

### 3.4. Listeria monocytogenes, Staphylococcus aureus, and Escherichia coli O157

Other pathogens monitored in accordance with the Provision of January 25, 2007, include *L. monocytogenes*, *S. aureus*, and *E. coli* O157. Although these pathogens were not found in the samples analyzed in this study, varying levels of prevalence have been reported in samples analyzed in Italy during official controls [17,18,19,20]. In particular, *L. monocytogenes* was found with a prevalence of 0.1% to 1.4%, coagulase-positive *Staphylococcus* ranged from 0.6% to 1.9%, and STEC was present at 1.3% to 1.6%. Given the observed prevalence rate of these pathogens in raw milk, it is crucial to understand their characteristics, potential sources of contamination, and the associated health risks.

*Listeria* spp. are ubiquitously present in the environment [79]; thus, raw milk contamination can occur at any stage of the production chain [80]. Even if rare, *L. monocytogenes* can also cause mastitis in cows, which may lead to direct contamination of milk [81].

*S. aureus* is one of the most common causes of bovine mastitis [82,83]. It produces enterotoxins (SEs) that may contaminate raw milk, posing a risk to human health [25]. SEs are heat-stable, so they are able to survive boiling [84]. Therefore, preventing raw milk contamination by SEs is crucial to avoid human disease [85]. It is important to note, however, that SEs are produced only when the bacterial concentration exceeds 10⁵ CFU/mL [86,87]. This may explain why the total absence of *S. aureus* in raw milk is not required [16]. The established minimum and maximum limits of 500 and 2000 CFU/mL, respectively, could in fact represent a compromise between the intention to avoid excessive restrictions, difficult to apply by producing farms, and the guarantee of an adequate level of safety for consumers.

*E. coli* O157, a pathogenic strain of *E. coli*, can contaminate raw milk either directly via feces or indirectly through contaminated feed and water [88,89]. It can produce Shiga toxins that can cause severe gastrointestinal symptoms and complications such as hemolytic uremic syndrome (HUS), particularly in vulnerable populations [90,91,92,93]. In 2022, 5011 confirmed cases of STEC infections were reported in the EU, with milk identified as a potential source of contamination [27]. The Provision of 25 January 2007 only addresses *E. coli* O157, but extending self-control strategies and official controls to include other harmful serogroups, such as O26, would improve the assessment of risks associated with raw milk consumption [35].

The presence of these microorganisms in raw milk could result from inadequate milking and post-milking practices, including unhygienic handling, improper transportation, and vending machines that are not adequately sanitized and maintained at appropriate temperatures to ensure product safety [89,94,95]. In this context, the adoption of strict hygiene protocols during milking and post-milking, as well as the implementation of effective biosecurity measures on farms, are essential [85].

### 3.5. Preventive Measures Applicable to Farmers

Compliance with the microbiological criteria established by the Provision of 25 January 2007 is essential to ensure a high level of public health protection. Indeed, raw milk contamination by *Campylobacter* spp., *Salmonella* spp., *E. coli* O157, *S. aureus*, *and L. monocytogenes* can pose serious risks to consumers [96]. Once raw milk exceeds the microbiological criteria (Table 1), it can no longer be intended for direct sale to consumers, making it crucial for farmers to adopt appropriate preventive measures to impede the presence of these pathogenic microorganisms. These measures include the implementation of rigorous biosecurity practices, both external and internal, along with proper milking hygiene management [78,97]. Indeed, it is essential to prevent wildlife and poultry from coming into contact with barns or milk storage areas, as they could represent a source of contamination for pathogens such as *Campylobacter* spp. and *Salmonella* spp., especially on farms where animals have access to an outdoor area [53,57,71]. In fact, as highlighted before, farms A, F, and G exhibited this issue (Table 2).

Additionally, it is crucial to pay attention to practices following milking. This includes maintaining the cold chain both on the farm and during transportation and storage at vending machines [98]. Proper cleaning and sanitization procedures must also be performed for the premises, equipment, and vehicles and containers used for transport. This applies to the milk dispenser at the vending machine as well, as established by the Provision of 25 January 2007. In this respect, it is essential for farmers to follow the manuals of good hygiene practices (GHP) approved by the Ministry of Health [99]. Despite some studies indicating that not all farmers fully understand the importance of these measures, they are fundamental for preventing the introduction and spread of pathogens on farms and for avoiding milk contamination [100,101]. Therefore, continuous training could be implemented for farmers on biosecurity practices, raw milk management, and the importance of adhering to the microbiological criteria established by legislation. Moreover, these measures are also relevant from an economic point of view. In fact, in the case of non-compliance, the marketing of raw milk must be suspended until the issue is resolved [16], which has a direct economic impact on the farmers. Finally, consumers also play a crucial role in their own safety. Although clear indications must be provided at points of sale and on the labels of milk bottles, recommending boiling the milk before consumption, it was reported that a portion of consumers do not follow this advice, exposing themselves to the risks associated with consuming raw milk [60,102,103]. In this regard, it may be necessary to repropose information campaigns to make the public aware of the risks associated with the consumption of raw milk and the importance of following the correct consumption instructions. However, even though consumers should adopt appropriate behaviors, farmers are still required to conduct self-monitoring to ensure that contaminated raw milk is not marketed [16].

## 4. Conclusions

In this retrospective study, the results of the analyses performed on 355 raw milk samples collected during official controls in Liguria between 2014 and 2023 for the detection of *Campylobacter* spp., *Salmonella* spp., *L. monocytogenes*, *S. aureus*, and *E. coli* O157 were analysed, to better characterize the associated risk for consumers. The samples were collected by the LHA services at vending machines of seven producing farms. Overall, six samples tested positive for *C. jejuni*, while only one sample tested positive for *S.* Veneziana. *L. monocytogenes*, *S. aureus*, and *E. coli O157* were never responsible for non-compliances. In farms where positive samples were detected, certain structural and/or operational non-compliances were identified, which may have contributed to milk contamination. Although consumers are required to boil raw milk before consumption, it is essential for farmers to implement all necessary measures to prevent milk contamination. In this regard, preventive measures are essential, both in limiting the introduction of pathogens into the farm and in avoiding direct or indirect contact of milk with contaminated sources. In this context, the role of the LHA is crucial in identifying such issues and promoting effective corrective actions, thereby ensuring a high level of safety for consumers.

## Figures and Tables

**Table 1:** Microbiological criteria for raw milk sold directly to consumers according to the Provision of 25 January 2007.

Microorganisms	Sampling Plan		Limits	
	n	c	m	M
*Staphylococcus aureus*	5	2	500 cfu/mL	2000 cfu/mL
*Listeria monocytogenes*	5	0	not detected in 25 mL	
*Salmonella* spp.	5	0	not detected in 25 mL	
*Escherichia coli* O157	5	0	not detected in 25 mL	
Thermotolerant *Campylobacter*	5	0	not detected in 25 mL	

n, number of units comprising the sample; c, number of sample units giving values between m and M; m, lower limit; M, upper limit.

**Table 2:** Details on the non-compliant samplings.

Year	Sampling Date	Producing Farm	Province	Potential Drivers of Contamination in Farms	Positivity
2016	5 September 2016	A	Genova	Proximity of the poultry house to the barn and milking area	*Campylobacter jejuni*
2016	12 May 2016	E	Genova	Poor management of the vending machine	*Campylobacter jejuni*
2016	21 November 2016	E	Genova	Poor management of the vending machine	*Campylobacter jejuni*
2017	13 June 2017	F	Genova	Proximity of the poultry house to the barn and milking area	*Campylobacter jejuni*
2017	16 October 2017	G	Genova	Improper cleaning of the post; incorrect straw bedding placement. The access to the barn and the milk storage area were not protected by barriers. The proximity of poultry area to the barn.	*Salmonella* Veneziana
2018	22 October 2018	E	Genova	Poor management of the vending machine	*Campylobacter jejuni*
2023	6 May 2023	A	Genova	Proximity of the poultry house to the barn and milking area	*Campylobacter jejuni*

## Data Availability

Data are available upon request.

## Supplementary Materials

The following supporting information can be downloaded at: https://www.mdpi.com/article/10.3390/ani15020286/s1, Table S1: Mandatory information to consumers according to the Provision of 25 January 2007; Table S2: Number of raw milk vending machines in Liguria (North-west Italy) from 2014 to 2023.
