# Supplementary material for: Microbiological Analysis Conducted on Raw Milk Collected During Official Sampling in Liguria (North-West Italy) over a Ten-Year Period (2014–2023)
Source: Animals (Basel). 2025 Jan 20;15(2):286. doi: 10.3390/ani15020286 (PMC11763045; doi:10.3390/ani15020286)
Supplement: Supplementary file 1 [file animals-15-00286-s001.zip › animals-3359866-supplementary.pdf]

Table S1. Mandatory information to consumers according to Provision of January 25, 2007.

Mandatory Information	Vending machine	Label
denomination of sale "raw milk from ... [specify species of animal]"	✓	✓
the date of milking	✓	
the date on which the milk was delivered to the dispenser	✓	
the date by which the milk must be consumed (to be consumed day/month/year)*	✓	✓
the business name of the production farm with the full address of the location	✓	✓
instructions for storing (0 °C and +4 °C)	✓	✓
net quantity (in liters)		✓
date of packaging (day/month/year)		✓

*:within three days from the date of milking (Ministry of Labor, Health and Social Policy ordinance of December 10, 2008, Ministry of Health decree of December 12, 2012).

Table S2. Number of raw milk vending machines in Liguria (North-west Italy) from 2014 to 2023.

Producing farms	Vending machines	2014	2015	2016	2017	2018	2019	2020	2021	2022	2023
A	A1		✓	✓							
	A2		✓	✓							
	A3		✓	✓							
	A4			✓							
	A5						✓	✓	✓	✓	✓
B	B1	✓	✓	✓	✓	✓	✓	✓	✓	✓	✓

	B2	✓	✓	✓	✓	✓	✓	✓	✓	✓	✓
C	C1		✓	✓							
D	D1		✓	✓	✓	✓	✓	✓	✓	✓	✓
E	E1		✓	✓	✓	✓	✓	✓			
	E2		✓	✓	✓	✓	✓	✓	✓	✓	✓
	E3		✓	✓	✓	✓					
	E4		✓	✓	✓	✓	✓	✓	✓	✓	✓
	E5		✓	✓	✓	✓	✓				
F	F1		✓	✓	✓	✓	✓				
G	G1			✓	✓	✓	✓	✓	✓	✓	✓
	G2				✓	✓	✓				
**TOTAL**		2	13	15	11	11	11	8	7	7	7
